# Screening of urine identifies *PLA2G16* as a field defect methylation biomarker for prostate cancer detection

**DOI:** 10.1371/journal.pone.0218950

**Published:** 2019-06-24

**Authors:** William E. Jarrard, Adam Schultz, Tyler Etheridge, Shivashankar Damodaran, Glenn O. Allen, David Jarrard, Bing Yang

**Affiliations:** 1 Carbone Comprehensive Cancer Center, University of Wisconsin, Madison, WI, United States of America; 2 Department of Urology, University of Wisconsin School of Medicine and Public Health, Madison, WI, United States of America; Sapporo Ika Daigaku, JAPAN

## Abstract

**Background:**

Prostate cancer (PC) is a multifocal disease. DNA methylation alterations are not restricted to the immediate peritumor environment, but spatially widespread in the adjacent and distant histologically normal prostate tissues. In the current study, we utilized high-throughput methylation arrays to identify epigenetic changes in the urine from men with and without cancer.

**Design, setting, and participants:**

DNA urine samples were enriched for methylated fragments using MBD methyl-binding antibodies and applied to high density CytoScanHD arrays. Significant loci were validated using quantitative pyrosequencing and binary logistic regression modeling applied to urine sample analyses in a training (n = 83) and validation approach (n = 84). Methylation alterations in prostate tissues using pyrosequencing at the *PLA2G16* locus were examined in 38 histologically normal specimens from men with (TA, n = 26) and without (NTA, n = 12) cancer and correlated to gene expression.

**Results:**

Methylation microarrays identified 3,986 loci showing significantly altered methylation in the urine samples from patients with PC compared to those without (TA vs NTA; p<0.01). These loci were then compared against subjects with their prostates removed to exclude non-prostate cell markers yielding 196 significant regions. Multiple CpGs adjacent to *PLA2G16* CpG island showed increased methylation in TA compared to NTA (p<0.01) in a large validation study of urine samples. The predictive accuracy of *PLA2G16* methylation at CG2 showed the highest predictive value at 0.8 (odds ratio, 1.37; 95% confidence interval, 1.16–1.62; p<0.001). Using a probability cutoff of 0.065, the sensitivity and specificity of the multivariate model was 92% and 35%. When histologically normal prostate tissues/biopsies from patients with PC (TA) were compared to subjects without cancer, significant hypermethylation of *PLA2G16* was noted (odds ratio, 1.35; 95% confidence interval, 1.07–1.71; p = 0.01).

**Conclusion:**

*PLA2G16* methylation defines an extensive field defect in histologically normal prostate tissue associated with PC. *PLA2G16* methylation in urine and prostate tissues can detect the presence of PC.

## Introduction

Urine is an underutilized source of PC biomarkers due to the presence of prostate epithelial cells [1]. Recent studies have evaluated various biomarkers in the urine including *PC3* [2], the only FDA-approved test, *TMPRSS2-ERG* [3, 4], DNA methylation of various genes [5–7], and exosomes and miRNAs [8, 9]. The urine presents an excellent source for PC biomarkers given the direct access to the prostate through the prostatic ducts, the concentration of any biomarker, and the noninvasive ease of collection.

PC development and progression are driven by the interplay of genetic and epigenetic changes including DNA methylation [10]. DNA methylation has advantages in cancer diagnosis because DNA is more stable than RNA and does not demonstrate autocatalytic capabilities. Traditional studies have focused on examining molecular alterations in tumor tissue and comparing this to adjacent non-tumor tissue [11, 12]. Our work has demonstrated that an extensive DNA methylation field effect exists across histologically normal prostate tissues that gives rise to the multifocality of cancer and its increased incidence with aging [13, 14]. These field defect methylation changes in non-cancer prostate tissues are an understudied area for urine biomarker development.

Overcoming low sensitivity is still an obstacle for urine tests in part due to the infrequent presence of cancer cells in the urine [15]. These cells are generally only found in higher volume cancers at a detection rate of less than 20% with cytopathology. Hypermethylation of GSTP1 was confirmed in the urine samples of patients with PC with a sensitivity between 21.4% and 38.9% that improved by prostatic massage [16]. One putative advantage of detecting non-tumor cells that contain a DNA methylation defect is the more frequent presence, which potentially generates a higher sensitivity.

To approach improved methylation markers in the urine, we employed a screening CytoScan HD microarray to compare urine from patients with and without cancer. This approach identified *PLA2G16* (*HRASLS3*), a class II tumor suppressor that exhibits promoter methylation and reduced expression levels in nasopharyngeal cancers [12, 17]. We find methylation alterations within *PLA2G16* are not limited to cancer, but also in normal prostate tissue from men with cancer. This gene a field defect in histologically normal prostate tissues associated with PC [13] and is useful in detecting cancer in the urine.

## Materials & methods

### Clinical samples

Three sources of samples were used for this study collected between 2013 and 2016. For CytoScanHD microarray scanning, six urine samples were obtained from patients with negative biopsies, five were from patients with aggressive PC (Grade Group, GG ≥4), and four were from patients post-prostatectomy. For validation, 167 urine samples were collected at the time of prostate biopsy. Patients with infection were excluded. Ninety samples were collected from patients with a positive biopsy for PC, termed tumor associated (TA, mean 65 yrs, GG ≥1), and 77 were from patients without PC termed non-tumor associated (NTA, mean 64 yrs). The NTA urine samples were from patients who all had at least two negative biopsy results for PC. Twelve urine samples from patients collected during a clinic visit after prostatectomy were used as controls (mean 59 yrs). **Table 1** documents the clinicopathologic characteristics for these patients. The University of Wisconsin Institutional Review Board has approved utilization of all the urine and tissue samples in this study, and written and informed consents have been obtained from all patients. None of the patients in this study received hormonal or chemotherapy.

**Table 1:** Urine sample clinical and pathologic characteristics.

	NTA	TA	Total	*p*-value
**Patients, n**	77	90	167	
**Age [yr]**	63.6 [35–74]	64.8 [45–81]	63.8 [35–81]	0.27
**PSA [ng/mL]**	7.7 [0.5–18.6]	13.1 [0.5–150]	10.6 [0.5–150]	**0.03**
**Prostate Size[g]**	51.4 [17–121]	42.5 [14–103]	46.5 [14–121]	**0.01**
**PSA Density [ng/mL/g]**	0.16 [0.01–0.64]	0.36 [0.02–5.40]	0.27 [0.01–5.40]	**0.01**
**Number of Cores Involved**	—	4 [1–12]	4 [1–12]	
**Max Core Involvement**	—	45% [1%-100%]	45% [1%-100%]	
**Ethnicity**				
**Caucasian**	88% [68/77]	98% [88/90]	93% [156/167]	
Family History*				
**Positive**	32% [25/77]	37% [33/89]	35% [58/166]	
**DRE**				
**Positive**	13% [10/77]	16% [14/90]	14% [24/167]	
**Grade Group**				
**1**	—	31	31	
**2**	—	24	24	
**3**	—	16	16	
**4**	—	14	14	
**5**	—	5	5	

*Some samples are missing data, data shown as mean and range, t-test, *p* value

Tissue was used as a second source obtained from OCT (Optimal cutting temperature compound) flash-frozen specimens. Non–tumor-associated (NTA, mean, 60 yrs) normal prostate tissues were obtained from 12 cystoprostatectomies after extensive histologic evaluation to exclude cancer. These specimens have no cancer present within the prostate. To define the relationship of methylation to tumor foci, histologic sections containing both cancer and normal regions were generated from 26 radical prostatectomy specimens (mean, 58 yrs). Sixteen patients were GG ≥2, and ten were GG ≥4. clinicopathologic characteristics have been provided previously [13]. Microdissection was performed to obtain tumor (T), normal tissue adjacent to tumor foci (2 mm, TAA), and normal tissue at a greater distance to tumor foci (10 mm, TAD).

A final source of specimens was from formalin fixed—paraffin embedded (FFPE) prostate biopsy tissue blocks that were obtained from two sets of patients (**S1 Table)**. The first group, non-tumor associated (NTA, n = 28), were men with two or more consecutive negative biopsies within 24 months. The second group, tumor associated (TA, n = 28), were from subjects diagnosed with PC who underwent radical prostatectomy, and final pathology was available. On final pathology, all cancer samples were GG ≥2. Four biopsy blocks were requested from each patient, H&E stained, and reviewed by a fellowship trained genitourinary pathologist. Samples with evidence of atypical small acinar proliferation and severe inflammation were excluded.

### DNA and RNA isolation

Urine samples were centrifuged to generate a pellet, and genomic DNA from the pellet was purified using a kit from IBI (Valley Park, MO), protocol: dx.doi.org/10.17504/protocols.io.3awgife. For frozen prostate tissues, ~2mg of tissue was homogenized and two-thirds of the sample was utilized for RNA isolation using Perfect Pure RNA Tissue Kit (5-Prime). DNA extraction was performed with a DNeasy Blood & Tissue Kit (Qiagen, MD). For the FFPE prostate biopsy samples, two 10-micron sections were obtained and DNA isolation and sodium bisulfite modification were performed using the EpiTect Plus FFPE Bisulfite Kit (Qiagen, MD), protocol: dx.doi.org/10.17504/protocols.io.3bfgijn. DNase was applied to total RNA to eliminate any contaminating genomic DNA.

### Methylated DNA immunoprecipitation and array profiling (MeDIP-chip)

The MeDIP-chip approach was adapted from previous studies utilizing methylated DNA immunoprecipitation followed by application to an Affymetrix copy number array [18, 19]. For each urine DNA sample, 0.5ug of genomic DNA was digested with Nsp1 at 37°C for 2 hours followed by adapter ligation at 16°C for 16 hours. Adapter-ligated DNAs were purified using an Amicon Ultra-centrifugation filter YM-100. An aliquot was saved as an input DNA, the rest of purified DNA was then denatured to single strands, and immunoprecipitated with 5ug of anti-5-methylcytosine antibody (Zymo Research) in IP buffer overnight at 4°C with rotation. Antibody-DNA complexes were captured with Protein A/G magnetic beads (Millipore, Massachusetts) at 4°C for 2 h. After washing, DNA was eluted from the beads with 50 uL TE buffer. Genome amplification of elutes, fragmentation, array hybridization, and array scanning were performed according to manufacturer’s instructions.

### DNA methylation

Bisulfite-modified DNA was amplified using PCR with one primer-biotinylated. The PCR products were confirmed with 2% agarose gel. The biotinylated PCR products were captured with streptavidin sepharose beads, denatured to single strand, and annealed to the sequencing primer for the pyrosequencing assay. Human Premixed Calibration Standard with different percentage of methylation was used as controls in each run. Methylation was quantified with the PyroMark MD Pyrosequencing System (protocol: dx.doi.org/10.17504/protocols.io.3bggijw) within the linear range of the assay. All samples were analyzed by three independent experiments. Primer sequences are available upon request.

### mRNA expression

Using frozen OCT samples from radical prostatectomy samples, cDNA was synthesized with qScript cDNA superMix (Quanta Biosciences) and PCR performed as described previously [20]. These primer sets are available upon request. To further eliminate gDNA contamination, the amplification of cDNA are achieved by designing exon-primed intron crossing primers. GAPDH was used as a control.

### Statistical analyses

For MeDIP-chip, CEL files generated from the scanned array image files by Affymetrix GeneChip Command Console Software were processed using Affymetrix Power Tools. Background subtraction and RMA normalization were performed to obtain normalized log2 transformed raw intensity values. Input subtraction was performed for each IP-input pair for normalization of copy number differences. Statistical analysis was performed on input subtracted values using Limma (R; Bioconductor). Using Affymetrix Cytoscan HD probe annotation data, we matched array probes to their predicted NspI digested fragments to predict enrichment regions. The analysis was restricted only to those fragments containing CpG sequences, and we considered p-values < 0.01 as significant.

The probe regions identified by microarray and expanded region ±1kb were validated using quantitative pyrosequencing using a larger sample size. The methylation at each CpG site was expressed as a percentage. All samples were run in duplicate (three independent experiments) and the two values were averaged. For each patient, the mean values for CG were calculated by averaging the methylation of the four biopsy tissue blocks for each patient. A two-tailed t-test was performed to analyze the significant differences between NTA and TA groups. Correlation between methylation and expression was performed by R-Pearson. For methylation, all CGs which significantly differentiated NTA from TA (p <0.05) were entered into a binary logistic regression model to test their ability to predict the presence of cancer. For urine samples, the performance of *PLA2G16* methylation was analyzed with binary logistic regression using Stata. The urine samples were randomly divided into training and validation groups. Univariate analysis of each CG was performed, areas under the curve (AUC) with p-values were calculated to assess the predictive value of detecting cancer. A multivariate model was also performed incorporating the following covariates: logPSA and the methylation marker. Age was not significant in the multivariate model, and did not improve the AUC of the model, therefore age was not included. The analysis for biopsy samples was same except the samples were not divided into training and validation groups due to the limitation of sample number.

## Results

### Identification of differential methylation in urine samples with cancer using CytoScanHD_arrays

Urine samples were compared from five patients with cancer (TA) and six with no cancer (NTA). DNA from a non-prostatic source (e.g. kidney, bladder) was evaluated by collecting and analyzing four subjects whose prostates had been removed. High-density CytoScanHD arrays were utilized to provide unbiased genome-wide coverage (> 2.4 million markers) and have an advantage in that they do not focus on specific genes or only promoter regions. Samples were enriched for methylated fragments as previously described using a methyl-binding antibody [19]. Microarray raw data and processed data has been deposited on ArrayExpress, accession number E-MTAB-7732. Methylation microarrays identify 3,986 probes, at 1,464 distinct gene regions, showing significantly altered methylation in the urine samples from patients with PC compared to those without (TA vs NTA: p<0.01) (**S1 Fig**). These loci were compared against subjects with their prostates removed to exclude non-prostate cell markers leaving 196 regions with methylation differences. This yielded 176 identified genes associated with cells of prostate origin, 9 long intergenic non-protein coding RNA and 11 uncharacterized probes (**S2 Table).** Attention was subsequently focused on *PLA2G16*, a biologically interesting gene, with clear methylation changes for validation of this approach.

### Validation of methylation within PLA2G16 in urine samples containing cancer

**Fig 1:**
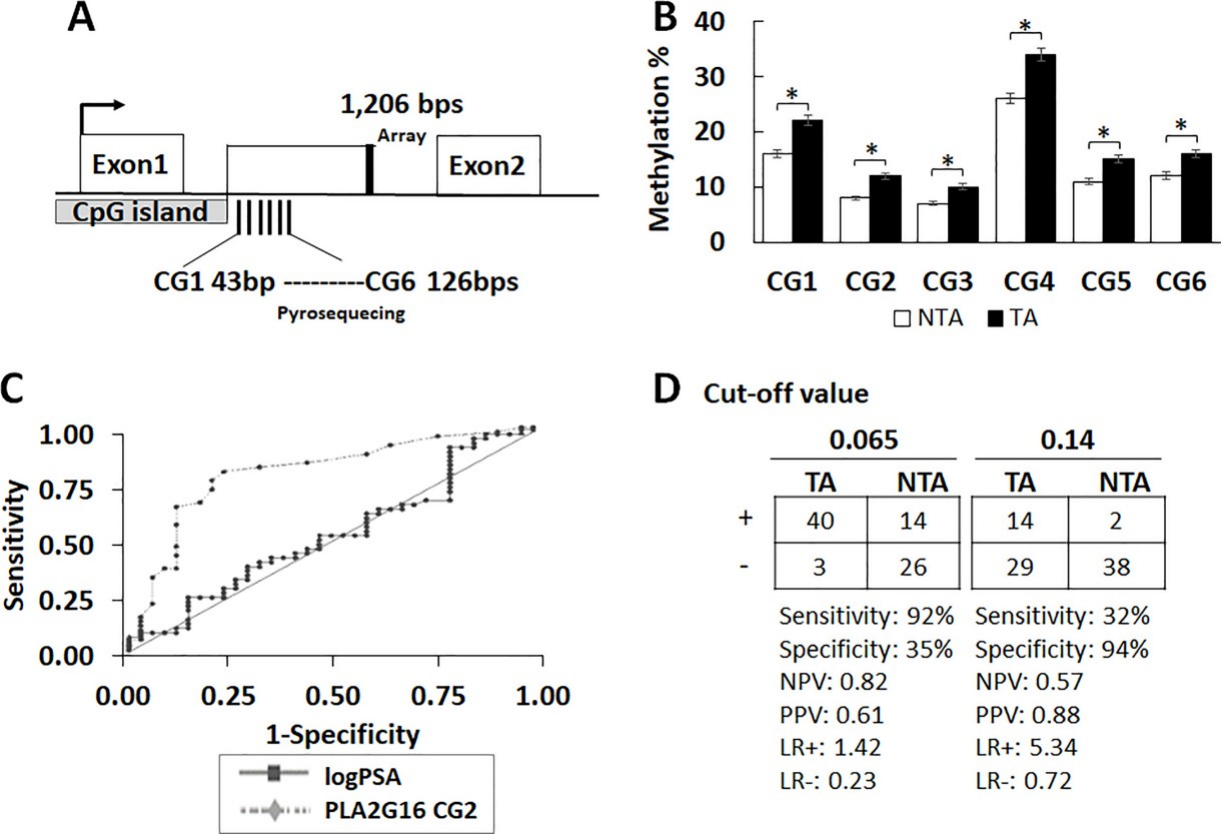
Methylation of *PLA2G16* increases in urine samples from patients with cancer. **A:** Location of the *PLA2G16* regions where DNA methylation was significantly altered in CytoScanHD microarray as well as detected by pyrosequencing after screening urine samples from patients with and without PC. The array probe is downstream 1,206 bps from the *PLA2G16* CpG island, and pyrosequencing confirms the region to be downstream 126-43bps from the *PLA2G16* CpG island, they both are within an intron. Six CGs were detected by pyrosequencing. **B:** *PLA2G16* methylation in urine samples analyzed using pyrosequencing. The values are from 3 individual experiments, shown as percentage Mean ± SEM. All loci shown were significantly altered in TA (tumor-associated, n = 90) when compared to NTA (non-tumor-associated, n = 77) samples. Student’s t-test was used to compare the differences between two groups, * p < 0.0006. **C:** ROC curves were generated to validate the predictive accuracy of the binary logistic regression models for discriminating TA and NTA urine samples. When used alone, *PLA2G16* CG2 (dashed curve) discriminated between patients with and without known cancer (AUC 0.798, p <0.001). LogPSA used alone in a univariate model shown (solid curve) AUC 0.519, p = 0.20. A multivariate model incorporating logPSA and CG2 had a lower predictive accuracy (AUC 0.790, 95% CI 1.16–1.63, p = 0.001, curve not shown here) for discriminating TA vs NTA. **D:** *PLA2G16* CG2 univariate model sensitivity, specificity, positive predictive value (PPV) and NPV. Two optimal cutoffs for predicted probabilities were used to define positive (+) and negative (−) test results.

Probes around the *PLA2G16* CpG island and expanded region (± 1kb) showed altered methylation. To further define and quantitate methylation within this region we used quantitative pyrosequencing across the identified area in a validation series of 167 urine samples (**Fig 1A)**. Clinicopathologic characteristics of this group are identified in **Table 1**. Methylation was significantly higher at all examined loci in the urine samples from TA compared to NTA (p<0.0006) within the *PLA2G16* region (**Fig 1B**; **S3 Table**).

The predictive accuracy of *PLA2G16* methylation was assessed in the 167 patient urine samples using binary logistic regression modeling. Patients were randomly assigned to training (n = 83, NTA 43, TA 40) and validation sets (n = 84, NTA 34, TA 50). When used in a univariate model, each CG had excellent predictive accuracy in the training sets of AUCs ranging from 0.621 to 0.717 (**Table 2)**. The generated model in the training set was then validated in the remaining 84 patients. *PLA2G16* CG2 showed the highest predictive value at 0.798 (p<0.001; **Fig 1C**). In contrast, logPSA demonstrated a predictive value of AUC = 0.52. Using a predicted probability cutoff of 0.065, the sensitivity and specificity of the multivariate model was 92% and 35%, respectively **(Fig 1D).** Using a cutoff of 0.14, the sensitivity and specificity of the univariate model was 32% and 94%, respectively. Urine methylation showed no significant difference between indolent and aggressive PC samples (GG 1& 2 vs GG3, 4 & 5; p>0.30).

**Table 2:** Odds of prostate cancer with PLA2G16 methylation alone or with clinical factors in urine.

Model Type	PLA2G16 CG	OR. (95% CI)	AUC	*p*-value
**Univariate**				
**(Training)**	PLAG (CG1)	1.15 (1.06–1.24)	0.699	0.001
	PLAG (CG2)	1.33 (1.13–1.57)	0.717	0.001
	PLAG (CG3)	1.16 (1.01–1.33)	0.646	0.030
	PLAG (CG4)	1.09 (1.03–1.15)	0.703	0.002
	PLAG (CG5)	1.09 (1.00–1.20)	0.640	0.046
	PLAG (CG6)	1.06 (0.99–1.14)	0.621	0.065
**Univariate**				
**(Validation)**	PLAG (CG1)	1.12 (1.04–1.21)	0.727	0.003
	PLAG (CG2)	1.37 (1.16–1.62)	0.798	<0.001
	PLAG (CG3)	1.29 (1.09–1.54)	0.744	0.004
	PLAG (CG4)	1.09 (1.03–1.15)	0.737	0.001
	PLAG (CG5)	1.17 (1.05–1.31)	0.756	0.005
	PLAG (CG6)	1.13 (1.04–1.23)	0.738	0.006
**Multivariate**			0.790	
**(Validation)**	PLAG (CG2)	1.38 (1.16–1.63)		<0.001
	logPSA	2.83 (0.56–14.37)		0.209

OR: odds ratio.

### PLA2G16 methylation increases in non-tumor prostate tissue marking a field of methylation change in cancer patients

**Fig 2:**
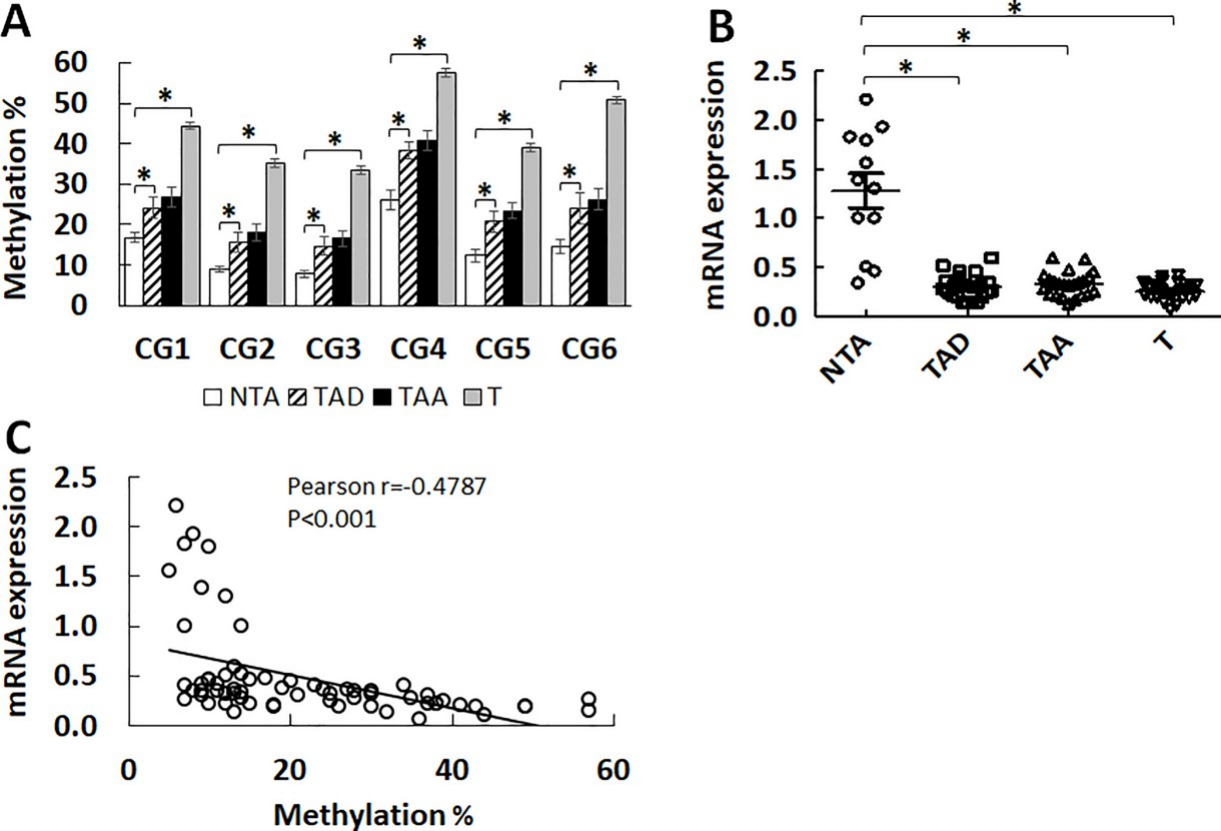
Methylation of *PLA2G16* increases in tumor and normal prostate tissue from patients with PC. **A:** The DNA methylation of *PLA2G16* in prostatectomy specimens was compared between NTA (non-tumor-associated, n = 12) patients without cancer, T (tumor, n = 26), TAA (tumor-associated adjacent, n = 26), and TAD (tumor-associated distant, n = 25). The tumors had the highest percent methylation. Significant differences in methylation between the NTA and T, TAA, and TAD were noted. The graph shows Mean ± SEM, one way Anova *p<0.05 for TAD or T when compared to T. **B:** *PLA2G16* gene expression was evaluated using RNA from the same samples. Significantly decreased expression (relative expression value X100) was observed in all T, TAA, and TAD than NTA. The values in both A and B are from 3 individual experiments, shown as Mean ± SEM, one way Anova, *p<0.05. **C:** Correlation between *PLA2G16* methylation and gene expression was analyzed using Pearson, the graph presents CG2 methylation vs expression (X100) in all NTA, T, TAA and TAD samples.

Methylation in this *PLA2G16* region was assessed in 26 tumor and matched non-tumor prostate tissues, as well as 12 tissues from men without PC. Tumor (T) showed more robust methylation than adjacent normal tissue (TAA) and distant normal tissue (TAD) for the 6 detected CGs (**Fig 2A, S3 Table**). However, no significant difference in the extent of methylation was seen between adjacent to tumor (TAA) and distant (TAD) normal tissue. When compared to NTA tissues (subjects without cancer), significant hypermethylation of *PLA2G16* was detected in all tissues (both nontumor and tumor) at all loci. Similar hypermethylation extent in both adjacent and distant normal tissues indicates that the epigenetic field defect in the prostate is spatially widespread and not localized solely to the immediate peritumor environment.

*PLA2G16* gene expression was evaluated using the same prostate tissues after prostatectomy. mRNA levels were detected with qRT-PCR using intron crossing primers and then normalized using GAPDH. **Fig 2B** shows the relative expression in prostate tissues for individual samples, *PLA2G16* was found to decrease 3–5 fold in the T and TAA/TAD groups compared to NTA (p <0.01). We analyzed the correlation between hypermethylation at each individual CpG and mRNA expression in prostate tissues and demonstrate a significant negative correlation to mRNA expression (R = -0.4608 to -0.5686, p <0.001) (**Fig 2C,** CG2).

### PLA2G16 methylation increases in histologically non-tumor biopsies from subjects with cancer compared to non-cancer

**Fig 3:**
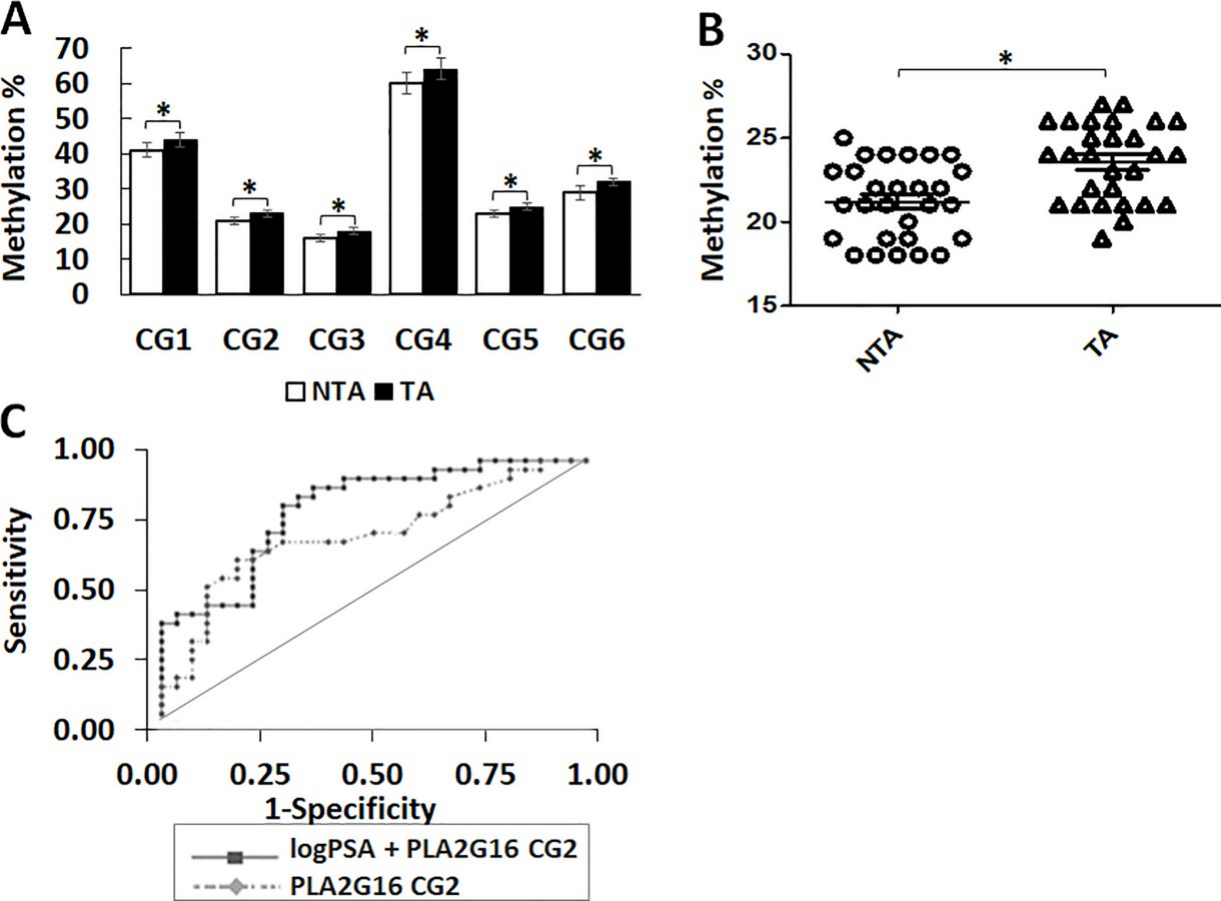
Methylation of *PLA2G16* increases in histologically normal biopsies from patients with PC. **A:** *PLA2G16* methylation for all detected CG loci in prostate biopsies. The values are from 3 individual experiments, shown as percentage Mean ± SEM. All loci shown were significantly altered in TA (n = 28) when compared to NTA (n = 28) samples, student’s t-test, * p<0.05. **B:** *PLA2G16* methylation for each individual samples at CG2 is shown, Mean ± SEM p = 0.0075 **C:** ROC curves were generated for a predictive accuracy of univariate and multivariate binary logistic regression models for discriminating TA and NTA prostate biopsies. When used alone, *PLA2G16* CG2 (dashed curve) discriminated between patients with and without known cancer (AUC 0.703, p = 0.01). A multivariate model incorporating logPSA and CG2 (solid curve) had higher a predictive accuracy (AUC 0.773, p = 0.0007) for discriminating TA vs NTA.

Formalin fixed tissue biopsy blocks were obtained from a study cohort and clinical data is provided (**S1 Table)**. NTA and TA groups were similarly matched except for PSA (7.5 vs 5.7; p = 0.012) and prostate size (47.3g vs 37.0g, p = 0.004). We averaged methylation of all 4 biopsy blocks from 3 individual experiments for all patients. Significant *PLA2G16* methylation differences between NTA and TA prostate biopsies are seen (p<0.05; **Fig 3A, S3 Table)**. Methylation for each individual patient at CG2 is detailed in **Fig 3B**. The predictive accuracy of *PLA2G16* methylation was assessed in prostate biopsies using a binary logistic regression models at individual loci. *PLA2G16* methylation discriminated between patients with and without cancer with an AUC ranging from 0.658 to 0.709 (**Table 3)**. A multivariate model incorporating *PLA2G16* methylation CG2 and logPSA performed with higher predictive accuracy (AUC 0.773, p = 0.0007) (**Fig 3C).** *PLA2G16* methylation did not differ between indolent and aggressive PC in the prostate biopsies.

**Table 3:** Odds of prostate cancer with PLA2G16 methylation alone or with clinical factors in prostate biopsies.

Model Type	PLA2G16 CG	OR. (95% CI)	AUC	p-value
**Univariate**	** **	** **	** **	** **
	PLAG (CG1)	1.12 (1.00–1.24)	0.668	0.040
	PLAG (CG2)	1.35 (1.07–1.71)	0.703	0.010
	PLAG (CG3)	1.44 (1.10–1.89)	0.709	0.007
	PLAG (CG4)	1.08 (1.01–1.15)	0.661	0.030
	PLAG (CG5)	1.25 (1.05–1.49)	0.658	0.010
	PLAG (CG6)	1.16 (1.02–1.32)	0.665	0.020
**Multivariate**			0.773	
	logPSA	0.10 (0.02–0.65)		0.016
	PLAG (CG2)	1.37 (1.06–1.75)		0.013

OR: odds ratio.

## Discussion

In this study, we identified through screening multiple genomic regions that demonstrate significantly altered methylation in the urine of patients who have PC. Urine samples from patients who no longer have prostates were utilized to exclude non-prostate changes. One significantly altered region at the *PLA2G16* gene was found not only to be hypermethylated in cancer tissue, but also in the histologically normal tissue of patients with cancer. This occurred at a unique CG island shore downstream from the *PLA2G16* promoter. This approach using markers that represent a field of methylation change in patients with PC may be more sensitive in detecting the disease because PC cells are extremely rare in patients with the disease [21–23].

PC is a multifocal disease. We have identified significant changes in *PLA2G16* DNA methylation not restricted to the immediate peritumor environment, but spatially widespread in the adjacent and distant histologically normal prostate tissues. *PLA2G16* methylation defines an extensive field defect in histologically normal prostate tissue associated with PC, which is consistent with the performance of other epigenetic biomarkers we have identified from tissue previously [13, 14]. This methylation alteration also permits a clear distinction between TA and NTA in both prostate biopsies and urine samples. The alteration detectable in prostate tissue and bodily fluid might be utilized clinically to reduce the need for repeat biopsy, which is associated with unnecessary costs and complications.

*PLA2G16* methylation revealed a reasonable predictive accuracy (AUC 0.8) from urine samples in the validation set. Larger methylation differences between TA vs. NTA subjects were observed in urine samples compared to prostate biopsies. Screening urine was utilized to identify this methylation alteration. One possibility for this is the cell of origin may be found at higher frequency in urine than in tissue perhaps related to increased shedding of abnormal epithelial cells. However, 8% of TA urine samples were negative possibly due to insufficient prostate cells present in their urine. The urine *PLA2G16* cutoff values can be adjusted to serve the intended purpose of the clinician. If the intent is to rule out any possibility of cancer, using a predicted probability cutoff of 0.14 yields 94% specificity. Conversely, if the intent is to detect more tumors, using a cutoff of 0.065 yields 92% sensitivity.

*PLA2G16* has been identified as a tumor suppressor gene in both breast cancer [12, 17, 24] and nasopharyngeal carcinoma [12]. Promoter hypermethylation of *PLA2G16* was found in 17% of nasopharyngeal cancer patients [12]. The aberrantly hypermethylated locus we have studied is located at a CpG shore (<0.2kb from *PLA2G16* promoter CpG island) which is spatially distinct from previous investigations. In general, hypermethylation of promoter CpG and their surrounding areas, called shores, is considered a hallmark of cancer and is believed to be involved in gradual silencing of tumor suppressor genes. In this study, we have identified significantly reduced *PLA2G16* expression in prostate tumor tissues, matched adjacent and distant normal prostate tissues compared to non-tumor associated prostate tissues, and it strongly correlates to DNA hypermethylation. Therefore, down-regulation of the tumor suppressor gene *PLA2G16* may play a role in multifocal prostate carcinogenesis. Conversely, high expression of *PLA2G16* has been reported to be associated with poor prognosis in non-small cell lung cancer patients and osteosarcomas suggesting its impact on tumor progression may be tumor type-specific [25, 26]. Sequencing for mutations in *PLA2G16* in these studies was not performed thus function was unclear. *PLA2G16* methylation was not altered by inflammation in the prostate (**S4 Table)**.

The current study has some limitations. Analysis of the association between higher GG and methylation was not significant. Therefore, *PLA2G16* methylation alone is not likely a powerful marker in differentiating indolent from aggressive PC. The current study has important implications for the early detection and development of PC since it does not rely on the presence of cancer cells to detect methylation differences. We speculate that methylation at this region might indicate those individuals at higher risk of having PC detected and further provide insight into the biology of the development of the disease. DNA methylation aberrations suggests that mechanisms, such as loss of the enhancer binding protein CTCF, might contribute to these early changes [27].

## Conclusions

The methylation of PLA2G16 when applied to histologically normal tissue distinguishes between patients who have cancer elsewhere in the gland and patients without. This methylation in normal appearing cells marks a field of susceptibility associated with the multifocal development of PC. PLA2G16 methylation differences are also detectable in urine samples that contain shed prostate epithelial cells. With further validation, these epigenetic markers may be utilized to improve the detection of prostate cancer.

## Supporting information

S1 Fig Identification of differential methylation in urine samples associated with PC using CytoScanHD microarrays. First 3,986 (a+b) probes showed significantly altered methylation between urine samples from patients with PC (TA, n = 5) and those without (NTA, n = 6), *p*<0.01. 6,998 (c+d) probes showed significantly differentiated methylation change between urine samples from patients without cancer (NTA) and those post prostatectomy, *p*<0.01. The overlap between a+b and c+d yield 196 probes were considered to be associated with PC. 176 out of 196 probes are associated with genes, 9 probes are LINC (long intergenic non-protein coding RNA) and 11 probes are LOC (uncharacterized).
(PDF)

S1 Table Clinical and pathologic characteristics of prostate biopsies. (PDF)

S2 Table The list of the probes showed significantly altered methylation in the urine samples associated with PC. (PDF)

S3 Table Methylation levels for all type of specimens. Methylation is shown as percentage Mean (SEM), t-test, p-value. Methylation for Prostatectomy tissues was analyzed by one-way Anova, p-value is not shown here.
(PDF)

S4 Table Methylation levels of samples containing inflammation and no inflammation in both urine and biopsy samples from the patients without PC. Methylation is shown as % Mean (SEM), t-test, p-value. *n is the slide number for biopsy specimens. Samples containing severe inflammation were excluded from analysis.
(PDF)
