# Supplementary material for: Screening of urine identifies PLA2G16 as a field defect methylation biomarker for prostate cancer detection
Source: PLoS One. 2019 Jun 24;14(6):e0218950. doi: 10.1371/journal.pone.0218950 (PMC6590820; doi:10.1371/journal.pone.0218950)
Supplement: S1 Fig — First 3,986 (a+b) probes showed significantly altered methylation between urine samples from patients with PC (TA, n = 5) and those without (NTA, n = 6), p<0.01. 6,998 (c+d) probes showed significantly differentiated methylation change between urine samples from patients without cancer (NTA) and those post prostatectomy, p<0.01. The overlap between a+b and c+d yield 196 probes were considered to be associated with PC. 176 out of 196 probes are associated with genes, 9 probes are LINC (long intergenic non-protein coding RNA) and 11 probes are LOC (uncharacterized). (PDF) [file pone.0218950.s001.pdf]

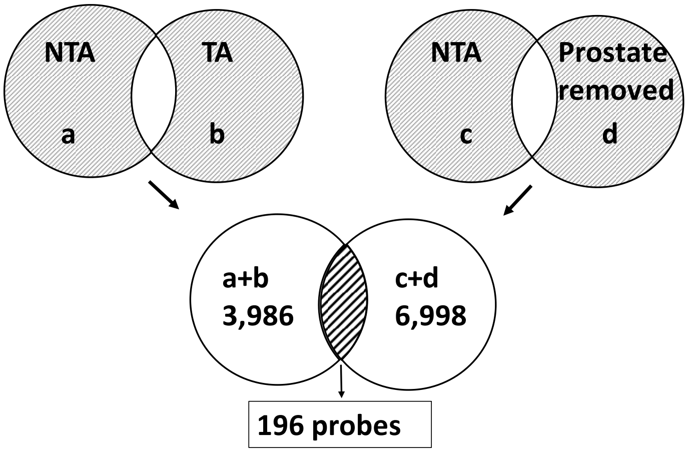

S1 Fig. Identification of differential methylation in urine samples associated with PC using CytoScanHD microarrays. First 3,986 (a+b) probes showed significantly altered methylation between urine samples from patients with PC (TA, n=5) and those without (NTA, n=6), $p<0.01$. 6,998 (c+d) probes showed significantly differentiated methylation change between urine samples from patients without cancer (NTA) and those post prostatectomy, $p<0.01$. The overlap between a+b and c+d yield 196 probes were considered to be associated with PC. 176 out of 196 probes are associated with genes, 9 probes are LINC (long intergenic non-protein coding RNA) and 11 probes are LOC (uncharacterized).
