# Supplementary material for: Screening of urine identifies PLA2G16 as a field defect methylation biomarker for prostate cancer detection
Source: PLoS One. 2019 Jun 24;14(6):e0218950. doi: 10.1371/journal.pone.0218950 (PMC6590820; doi:10.1371/journal.pone.0218950)
Supplement: S1 Table — (PDF) [file pone.0218950.s002.pdf]

S1 Table. Clinical and pathologic characteristics of prostate biopsies.

	NTA	TA	Total	p-value
**Patients, n**	28	28	56	
**Age [yr]**	59.9 [54-71]	61.1 [52-69]	60 [54-71]	0.35
**PSA [ng/mL]**	7.5 [4.0-15.0]	5.7 [3.4-10.6]	6.6 [4.0-15.0]	0.01
**Prostate Size [g]**	47.3 [25-85]	37.0 [22-56]	42.1 [22-85]	0.01
**PSA Density [ng/mL/g]***	0.17 [0.07-0.35]	0.16 [0.06-0.35]	0.17 [0.07-0.35]	0.68
**Ethnicity:**				
**Caucasian**	93% [26/28]	93% [26/28]	93% [52/56]	
**Family History:***				
**Positive**	21% [5/24]	37% [10/27]	29%[15/51]	
**DRE:***				
**Positive**	21% [6/28]	15% [4/27]	18% [10/55]	
**Grade Group:**				
**2**	---	19	19	
**3**	---	6	6	
**4**	---	2	2	
**5**	---	1	1	
**Clinical Stage:**				
**T2**	---	5	5	
**T2b**	---	4	4	
**T2c**	---	9	9	
**T3a**	---	8	8	
**T3b**	---	2	2	

*Some samples are missing data, data shown as mean and range, t-test *p*-value.
