# Supplementary material for: Screening of urine identifies PLA2G16 as a field defect methylation biomarker for prostate cancer detection
Source: PLoS One. 2019 Jun 24;14(6):e0218950. doi: 10.1371/journal.pone.0218950 (PMC6590820; doi:10.1371/journal.pone.0218950)
Supplement: S2 Table — (PDF) [file pone.0218950.s003.pdf]

S2 Table. The list of the probes showed significantly altered methylation in the urine samples associated with PC.

Probe	Chromosome	Probe location	Gene/other
C-4TGOM	16	16117773-16117798	ABCC1
C-7DOLY	10	1596062-1596087	ADARB2
C-6JFMA	8	26697540-26697565	ADRA1A
C-5ZTZV	1	36475162-36475187	AGO3
C-4JNFJ	6	90307445-90307470	ANKRD6
C-7DNEU	1	18011090-18011115	ARHGEF10L
C-4UQSS	10	123492295-123492320	ATE1
C-6ZHQU	12	51185621-51185646	ATF1
C-5DWGE	10	29275015-29275040	C10orf126
C-3UGMZ	7	7263291-7263315	C1GALT1
C-4AKEV	4	81549828-81549853	C4orf22
C-6DFJN	12	2794393-2794418	CACNA1C
C-4HLYI	7	44284945-44284970	CAMK2B
C-5OJXA	20	54594037-54594062	CBLN4
C-6POHM	1	208125816-208125841	CD34
C-4WEZV	16	82685029-82685054	CDH13
C-7DRFJ	18	62965440-62965465	CDH7
C-5KEOB	2	169584091-169584115	CERS6
C-7GSAS	X	85199164-85199189	CHM
C-4EBYR	17	7361108-7361132	CHRNB1
C-7OLQL	12	70704159-70704184	CNOT2
C-4UCJQ	2	124916612-124916637	CNTNAP5
C-5VLJY	10	105839297-105839322	COL17A1
C-5JAOH	2	62239437-62239462	COMMD1
C-4VYBW	3	148884208-148884233	CP
C-4BIEH	3	131361167-131361192	CPNE4
C-4FKMI	10	68019419-68019444	CTNNA3
C-7FRBI	1	69127351-69127376	DEPDC1
C-6PQCM	X	32553869-32553894	DMD
C-3HHDW	21	45673145-45673170	DNMT3L
C-4HCJO	8	105409972-105409996	DPYS
C-7DNGW	1	221708803-221708828	DUSP10
C-5QYCH	7	109624911-109624936	EIF3IP1
C-3XLMS	2	119520371-119520396	EN1
C-5IHWJ	3	90115218-90115243	EPHA3
C-4PNQJ	3	56275029-56275054	ERC2
C-7HOYF	6	82420096-82420121	FAM46A
C-7FTYI	1	150971486-150971511	FAM63A

C-6OQGH	5	107458750-107458775	FBXL17
C-3LRHY	13	102399184-102399209	FGF14
C-5PHRR	14	28867449-28867474	FOXG1
C-5HZBO	18	74960117-74960142	GALR1
C-6NENM	14	55331267-55331292	GCH1
C-6BXVV	18	11781329-11781354	GNAL
C-7LOTA	20	57500605-57500630	GNAS
C-4ZCME	14	66975971-66975996	GPHN
C-5XIZU	2	11753480-11753504	GREB1
C-7KOIK	X	122151091-122151116	GRIA3
C-5BPJG	9	124058542-124058567	GSN
C-7CSTO	6	139477637-139477662	HECA
C-3XDNX	X	29541877-29541902	IL1RAPL1
C-6RXGN	19	7185434-7185459	INSR
C-5SQQY	19	44199398-44199423	IRGC
C-5UNVL	21	35113863-35113888	ITSN1
C-5FJIS	18	44530234-44530259	KATNAL2
C-4LNRT	5	113376461-113376486	KCNN2
C-5KTUH	11	77736886-77736911	KCTD14
C-5QGQV	19	5050097-5050122	KDM4B
C-7QYGB	7	36363695-36363720	KIAA0895
C-5NDYN	8	29027537-29027562	KIF13B
C-5RIHX	5	154373898-154373923	KIF4B
C-7QHWN	12	52556676-52556701	KRT80
C-4HZCZ	21	31987500-31987525	KRTAP6-1
C-3CZMZ	6	108776790-108776814	LACE1
C-6SDDQ	21	26767084-26767109	LINC00158
C-3PZEV	2	14214967-14214992	LINC00276
C-3ETRF	13	31467800-31467825	LINC00545
C-5IRGK	5	39989671-39989696	LINC00603
C-5KXDT	3	106497214-106497239	LINC00882
C-6OXZE	15	98299234-98299258	LINC00923
C-4NCMG	1	88392571-88392596	LINC01364
C-7GQQQ	15	94602332-94602357	LINC01581
C-7EFIJ	9	28332661-28332686	LINGO2
C-3MGXV	11	133893258-133893282	LOC100128239
C-7LJMK	11	67094279-67094304	LOC100130987
C-4KSTO	19	29309750-29309775	LOC100420587
C-4KNAE	5	39667796-39667821	LOC101926940
C-6APXY	6	114486357-114486382	LOC101927768
C-6CEKJ	1	87727148-87727173	LOC101927844

C-6QJQW	X	4926439-4926464	LOC101928201
C-6MRVI	2	150704673-150704698	LOC101929231
C-4UPAZ	8	123487720-123487745	LOC105375734
C-6FIYQ	2	130629056-130629081	LOC389033
C-6RXYJ	1	13686908-13686933	LOC391003
C-7GZUU	5	121426184-121426209	LOX
C-3NHFS	3	14237753-14237778	LSM3
C-5JDGL	X	114531588-114531613	LUZP4
C-6MOIR	1	236063012-236063037	LYST
C-6MPZX	1	39931950-39931975	MACF1
C-3YMXP	X	51633555-51633580	MAGED1
C-5GSRU	18	13881868-13881893	MC2R
C-4NTGG	3	182782885-182782910	MCCC1
C-6BBBR	1	85490603-85490628	MCOLN3
C-3KSJI	7	116346152-116346177	MET
C-6MTAF	1	240857535-240857560	MIR1273E
C-4CXPW	13	41280808-41280833	MIR320D1
C-5TLJP	10	58707183-58707208	MIR3924
C-6FGZJ	14	65775503-65775528	MIR4708
C-7PIZS	9	114008028-114008053	MIR7702
C-5KLDJ	10	22020522-22020546	MLLT10
C-7GXIE	1	161278120-161278145	MPZ
C-4TGTZ	6	24410147-24410172	MRS2
C-3DBVZ	6	151281000-151281025	MTHFD1L
C-5JNLZ	15	52611507-52611532	MYO5A
C-6SJVD	3	175212038-175212063	NAALADL2
C-6VBOI	5	141510398-141510423	NDFIP1
C-6AHCD	4	170344003-170344027	NEK1
C-6SALG	18	77231058-77231083	NFATC1
C-4YUCB	5	36932001-36932026	NIPBL
C-3CHOF	12	52435326-52435351	NR4A1
C-5RIMR	10	84168095-84168119	NRG3
C-4RREC	15	76247962-76247987	NRG4
C-5LZLI	2	50502139-50502163	NRXN1
C-6HKMN	14	79613486-79613511	NRXN3
C-7AGLD	2	139477375-139477400	NXPH2
C-4YHJV	2	175048887-175048912	OLA1
C-5DUGB	19	15912858-15912883	OR10H1
C-7PNBX	3	31827227-31827252	OSBPL10
C-6TDWV	11	3191660-3191685	OSBPL5
C-3ODUA	14	57312314-57312339	OTX2

C-4CNIO	4	135449887-135449912	PABPC4L
C-3PSWW	X	49591772-49591797	PAGE4
C-7DFMH	10	55948490-55948515	PCDH15
C-5IPLI	7	82652180-82652205	PCLO
C-7HXOD	15	101969559-101969584	PCSK6
C-7OIYM	11	117076213-117076238	PCSK7
C-4APJF	2	183112723-183112748	PDE1A
C-7PKJZ	11	46059628-46059653	PHF21A
C-3PIOB	11	17183460-17183485	PIK3C2A
C-5YJGA	11	63379913-63379938	PLA2G16
C-4RTFY	10	96004421-96004445	PLCE1
C-6NNOI	16	81988103-81988128	PLCG2
C-6HXHA	1	242638142-242638167	PLD5
C-6XLRP	X	82783379-82783403	POU3F4
C-5EQWA	12	81840762-81840787	PPFIA2
C-4VAOL	12	27786162-27786187	PPFIBP1
C-5ZTNB	14	94732765-94732789	PPP4R4
C-5HZAD	3	169959145-169959170	PRKCI
C-5NVDF	10	53145271-53145296	PRKG1
C-7ESOO	1	107630522-107630547	PRMT6
C-4EWFG	9	8980680-8980705	PTPRD
C-4PFEG	4	122400737-122400762	QRFPR
C-7FGCN	14	68925340-68925365	RAD51B
C-6ZXZL	4	99339930-99339955	RAP1GDS1
C-7CNVE	1	211658667-211658692	RD3
C-5PBTC	10	62643265-62643290	RHOBTB1
C-5JFSC	X	73831777-73831802	RLIM
C-5ZYRK	3	125681759-125681784	ROPN1B
C-5ILYC	X	18681810-18681834	RS1
C-7IRPA	10	64013742-64013767	RTKN2
C-5ALKV	3	45781425-45781450	SACM1L
C-3FMIS	16	51200603-51200628	SALL1
C-5VBZR	3	159500565-159500590	SCHIP1
C-7KWZJ	2	167341049-167341074	SCN7A
C-5UELD	1	67888347-67888371	SERBP1
C-3SSRO	6	74314001-74314026	SLC17A5
C-5WCRJ	12	40400119-40400144	SLC2A13
C-6RHER	5	168372539-168372564	SLIT3
C-6QKCE	15	25313896-25313921	SNORD116-8
C-5OXCB	X	138563973-138563998	SRD5A1P1
C-3HEZE	8	134644380-134644405	ST3GAL1

C-6TYCE	3	186691260-186691285	ST6GAL1
C-4TFZR	5	32621626-32621651	SUB1
C-5DYHY	2	108900525-108900550	SULT1C2
C-7GEIC	14	64519506-64519531	SYNE2
C-6ETWK	16	69050068-69050093	TANGO6
C-7KJLG	X	123753057-123753082	TENM1
C-3HSMI	15	71547535-71547560	THSD4
C-6NRSX	11	86991968-86991993	TMEM135
C-7AWAQ	3	45247753-45247778	TMEM158
C-5ZPCO	7	129839671-129839696	TMEM209
C-5MTLY	4	69102518-69102543	TMPRSS11B
C-3NQIB	13	43208299-43208324	TNFSF11
C-5VSPZ	3	189455540-189455565	TP63
C-5KDNB	8	110132754-110132779	TRHR
C-6RBHB	16	31238009-31238034	TRIM72
C-6MNLD	20	51940542-51940567	TSHZ2
C-3RAUS	8	143483639-143483664	TSNARE1
C-3BWMD	15	76157068-76157093	UBE2Q2
C-6KXHN	6	149213124-149213149	UST
C-3FZUB	X	129091685-129091710	UTP14A
C-6DHNR	5	82821119-82821144	VCAN
C-5OSHL	X	8221672-8221697	VCX2
C-6AFHH	3	42531083-42531108	VIPR1
C-5VZMF	2	219757745-219757770	WNT10A
C-3DFOE	4	184185454-184185479	WWC2
C-3VPEG	16	78833588-78833613	WWOX
C-3AGSC	19	36202180-36202205	ZBTB32
C-6DOAQ	2	112988141-112988166	ZC3H8
C-5GQLA	3	28496513-28496538	ZCWPW2
C-3WLSU	15	80435691-80435716	ZFAND6
C-3ZYQA	2	180407255-180407279	ZNF385B
C-5RDRK	18	72384818-72384843	ZNF407
C-7PJSD	19	58919467-58919492	ZNF584
C-3UQWP	7	64019749-64019773	ZNF680
C-6YQFR	7	64836469-64836494	ZNF92
