# Supplementary material for: Screening of urine identifies PLA2G16 as a field defect methylation biomarker for prostate cancer detection
Source: PLoS One. 2019 Jun 24;14(6):e0218950. doi: 10.1371/journal.pone.0218950 (PMC6590820; doi:10.1371/journal.pone.0218950)
Supplement: S3 Table — Methylation is shown as percentage Mean (SEM), t-test, p-value. Methylation for Prostatectomy tissues was analyzed by one-way Anova, p-value is not shown here. (PDF) [file pone.0218950.s004.pdf]

S3 Table. Methylation levels for all types of specimens.

		**CG1**	**CG2**	**CG3**	**CG4**	**CG5**	**CG6**
**Urine**	NTA (n=77)	16 (0.6)	8 (0.3)	7 (0.4)	26 (1.0)	11 (0.6)	12 (0.7)
	TA (n=90)	22 (0.9)	12 (0.6)	10 (0.6)	34 (1.2)	15 (0.7)	16 (0.8)
	p-value	0.0000	0.0000	0.0002	0.0000	0.0004	0.0006
**Prostatectomy Tissue (OCT)**	NTA (n=12)	17(1.3)	9 (0.8)	8 (0.9)	26 (2.6)	12 (1.5)	15 (1.9)
	TAD (n=26)	24 (2.7)	15 (2.4)	15 (2.3)	38 (2.0)	21 (2.7)	24 (3.8)
	TAA (n=26)	27 (2.4)	18 (2.1)	17 (1.9)	41 (2.4)	23 (1.9)	26 (2.6)
	T (n=25)	44 (2.5)	35 (1.8)	33 (1.7)	57 (2.5)	39 (2.0)	51 (3.4)
		CG1	CG2	CG3	CG4	CG5	CG6
**Biopsy (FFPE)**	NTA (n=28)	41 (1.6)	21 (0.8)	16 (0.6)	58 (2.6)	23 (0.8)	29 (1.2)
	TA (n=28)	44 (1.9)	23 (1.0)	18 (0.9)	64 (2.9)	25 (1.0)	32 (1.5)
	p-value	0.0380	0.0075	0.0035	0.0253	0.0093	0.0155

Methylation is shown as percentage Mean (SEM), t-test, *p*-value. Methylation in prostatectomy tissues was analyzed by one-way Anova, *p*-value is not shown here.
