# Supplementary material for: Screening of urine identifies PLA2G16 as a field defect methylation biomarker for prostate cancer detection
Source: PLoS One. 2019 Jun 24;14(6):e0218950. doi: 10.1371/journal.pone.0218950 (PMC6590820; doi:10.1371/journal.pone.0218950)
Supplement: S4 Table — Methylation is shown as % Mean (SEM), t-test, p-value. *n is the slide number for biopsy specimens. Samples containing severe inflammation were excluded from analysis. (PDF) [file pone.0218950.s005.pdf]

S4 Table. Methylation levels of samples containing inflammation and no inflammation in both urine and biopsy samples from the patients without PC.

		**CG1**	**CG2**	**CG3**	**CG4**	**CG5**	**CG6**
**Urine**	No inflammation (n=48)	16 (0.6)	8 (0.3)	7 (0.4)	25 (1.1)	11 (0.7)	12 (0.8)
	Inflammation (n=29)	14 (1.1)	8 (0.6)	7 (0.7)	23 (2)	11 (1.2)	11 (1.4)
	p-value	0.21	0.63	0.54	0.42	0.63	0.67
**Biopsy**	No inflammation (*n=53)	41 (1.3)	22 (0.5)	16 (0.4)	58 (1.9)	23 (0.6)	29 (1.0)
	Inflammation (n=58)	41 (1.1)	21 (0.5)	16 (0.5)	58 (1.8)	23 (0.6)	28 (0.7)
	p-value	0.87	0.17	0.62	0.96	0.50	0.34

Methylation is shown as % Mean (SEM), t-test, p-value. *n is the slide number for biopsy specimens. Samples containing severe inflammation were excluded from analysis.
